# Supplementary material for: Optimizing Antimicrobial Peptide Design: Integration of Cell-Penetrating Peptides, Amyloidogenic Fragments, and Amino Acid Residue Modifications
Source: Int J Mol Sci. 2024 May 30;25(11):6030. doi: 10.3390/ijms25116030 (PMC11173194; doi:10.3390/ijms25116030)
Supplement: Supplementary file 1 [file ijms-25-06030-s001.zip › ijms-3009649-supplementary.pdf]

Table S1. Characteristics of the peptides from chromatography and mass spectrometry analysis

1.	R23F^{S}*	RKKRRQRRRGG-Sar-GVVVHI-X-GGKF-NH2
	Retention time	14.6 min
	Calculated monoisotopic mass	2613.5541
	Observed monoisotopic mass (ESI MS)	2613.5240
2.	V31K^{S}	VVVHINGGKFGGGGSRQIKIWFQNRRMKWKK
	Retention time	9.2 min
	Calculated monoisotopic mass	3610.9
	Observed monoisotopic mass (MALDI MS)	3611.7
3.	V31K^{S}*	G-VVVHINGGKFGG-Sar-GSRQIKIWFQNRRXKWKK-NH2
	Retention time	13.7 min
	Calculated monoisotopic mass	3663.1015
	Observed monoisotopic mass (MALDI MS)	3669.2
4.	R44K^{S}	RKKRRQRRRGGGGVVVHINGGKFGGGGSRQIKIWFQNRRMKWKK
	Retention time	10.4 min
	Calculated monoisotopic mass	5159.9
	Observed monoisotopic mass (MALDI MS)	5163.2
5.	R44K^{S}*	RKK-K-RQRRRGG-Sar-GVVVHINGGKFGG-Sar-GSRQIKIWFQNRRXKWKK-NH2
	Retention time	13.6 min
	Calculated monoisotopic mass	5141.0306
	Observed monoisotopic mass (ESI MS)	5141.0848

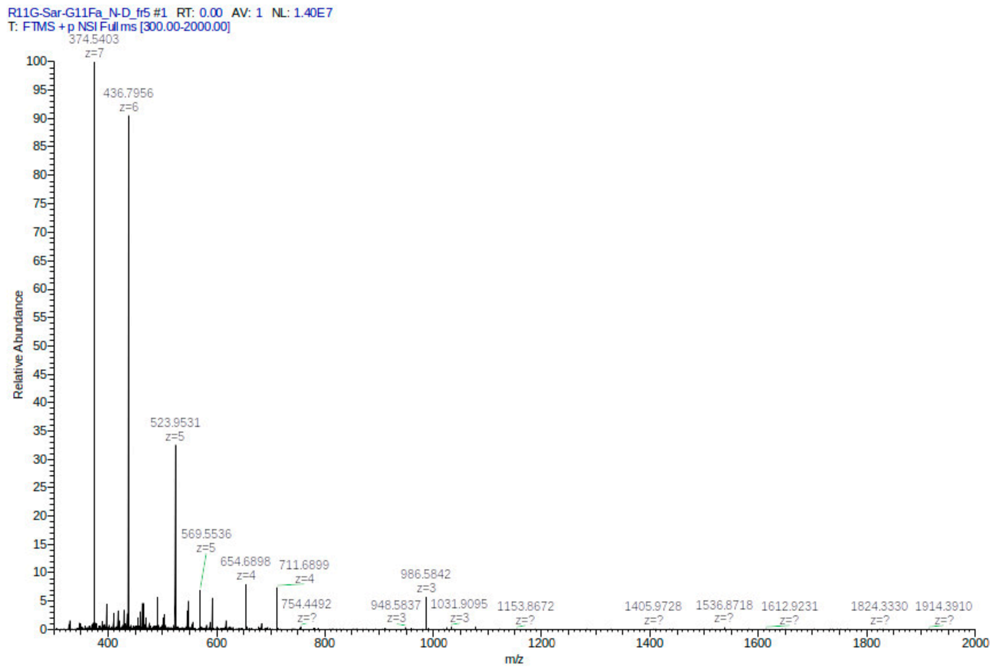

Figure S1. Mass spectrometry analysis for R23F^{S}*.

IQChem

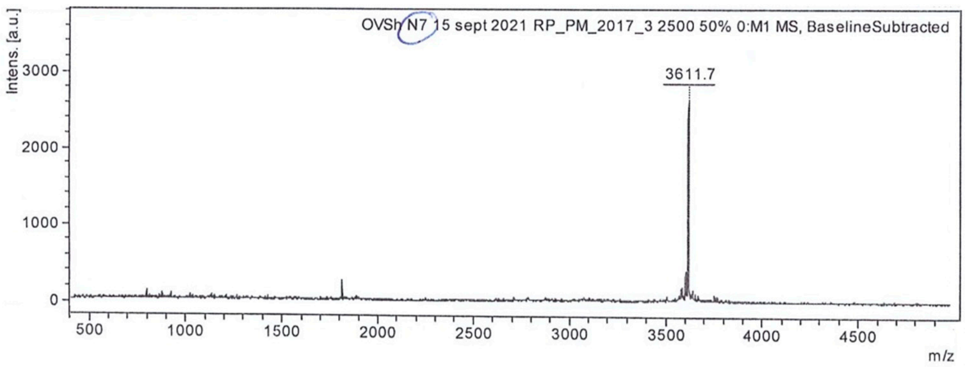

Figure S2. Mass spectrometry analysis for V31K^{S}.

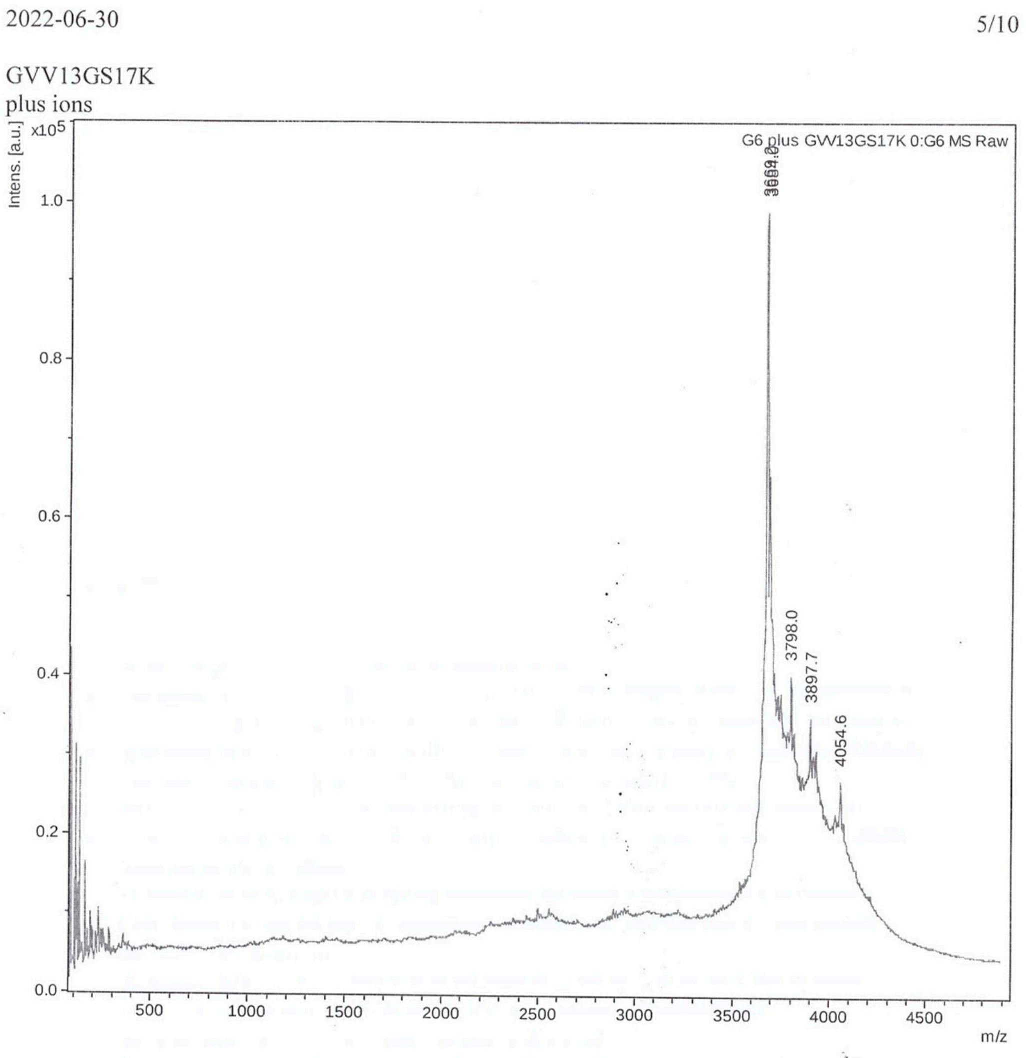

Figure S3. Mass spectrometry analysis for V31K^{S}*.

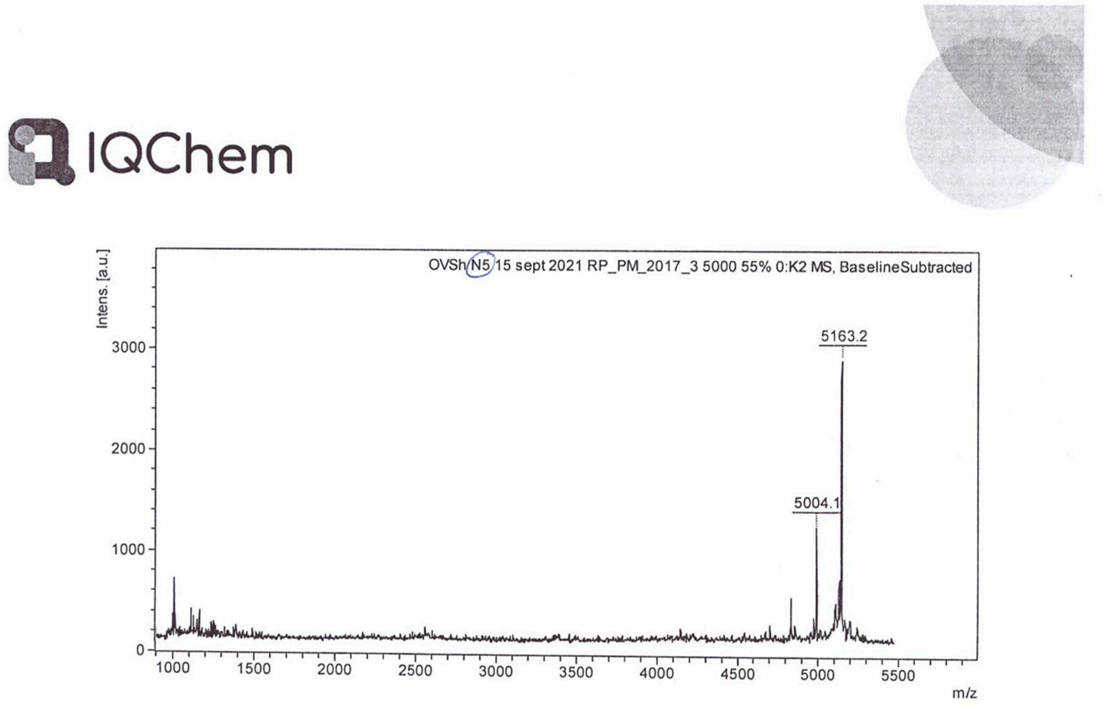

Figure S4. Mass spectrometry analysis for R44K^{S}.

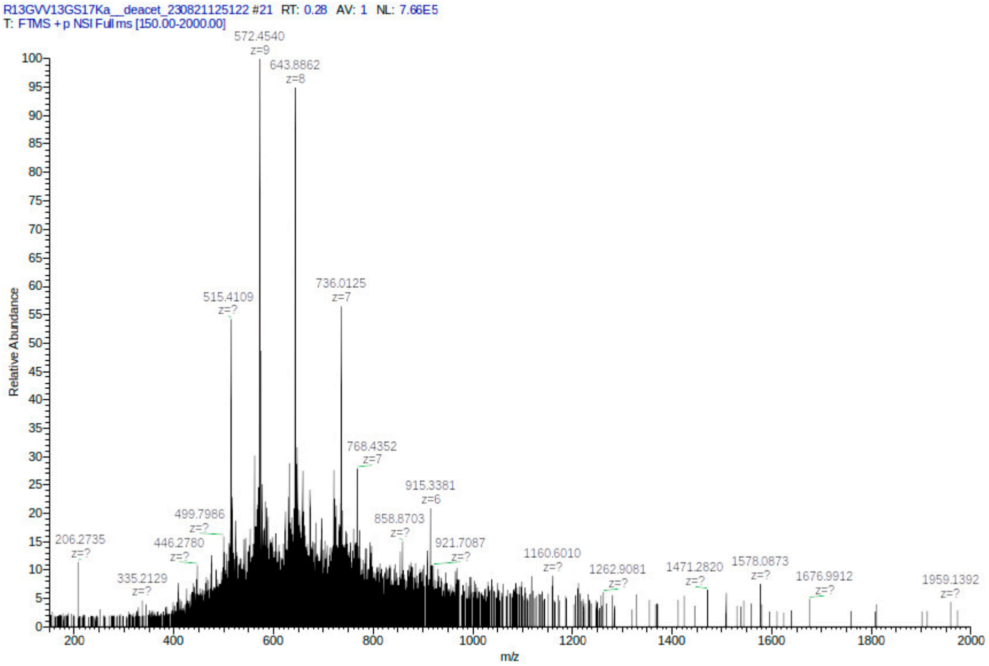

Figure S5. Mass spectrometry analysis for R44K^{S}*.
